# Lectures on the Principles and Practice of Physic

**Published:** 1847-12

**Authors:** 


*Lectures on the Principles and Practice of Physic.* Delivered at King's College, London, by Thomas Watson, M. D., Fellow of the Royal College of Physicians, late Physician to the Middlesex Hospital, and formerly Fellow of St. John's College, Cambridge. Third American from the last London edition. Revised with additions by Francis Condie, M. D., Secretary of the College of Physicians, author of a Treatise on Diseases of Children, etc. etc. Philadelphia: Lea & Blanchard. 1847. (From the Publishers.)

Commendation of these lectures would be only reiterating the often recorded opinion of the Profession. By universal consent the work ranks among the *very best* text books in our language. The author has no theories to defend, and indulges in few speculations; his work is therefore of the kind most needed, a *practical* one. The *style* is an additional recommendation, being easy and familiar, free from pedantic efforts to "show off" by the use of high sounding words, and far-fetched terms, which but confuse the student, and excite the risibles of the sensible practitioner. Doctor Watson is a perfect hater of technicals. His object is to impart a knowledge of disease and its cure, not of the best manner of so compounding words as to make them the most difficult of pronunciation, even though at the risk of an obscurity of meaning. He would make of his hearers and readers, physicians, not pedants. We commend his example to certain writers and lecturers we wot of this side the Atlantic. Dr. Condie has, in this edition, supplied some omissions which were very important, particularly to western practitioners and students. G. N. F.

